# Eversion and withdrawal of an intromittent organ before sexual maturation prepares male beetles for copulation

**DOI:** 10.1098/rsos.161029

**Published:** 2017-08-09

**Authors:** Yoko Matsumura, Takuya Kubo

**Affiliations:** 1 Laboratory of Systematic Entomology, Department of Ecology and Systematics, Graduate School of Agriculture, Hokkaido University, Sapporo 060-8589, Japan; 2 Department of Functional Morphology and Biomechanics, Zoological Institute, University of Kiel, Am Botanischen Garten 1-9, 24098 Kiel, Germany; 3 Graduate School of Environmental Earth Science, Hokkaido University, Sapporo 060-0810, Japan

**Keywords:** Chrysomelidae, Criocerinae, flagellum, genital evolution

## Abstract

Some species of criocerine beetles have a hyper-elongated part of the intromittent organ called a flagellum. In resting position, the flagellum is stored in a specialized internal sac in the intromittent organ. This specialized state of the flagellum and internal sac is indispensable during copulation for flagellar insertion into the female spermathecal duct for sperm transfer. However, the morphogenesis of the flagellum does not generate the active state of the flagellum; rather, the flagellum is generated in an inactive and completely coiled state. After eclosion, males of *Lema coronata* evert and withdraw the internal sac multiple times before sexual maturation, without mounting a female. This behaviour serves to uncoil the flagellum and guide it into the active state with the aid of surface structures on the internal sac. A closely related species, *Lema dilecta*, also has a long flagellum and undergoes the same behaviour to place the flagellum in the active position. However, some other species of criocerine beetles with much shorter flagella can attain the active state without exhibiting this behaviour. Based on a previously proposed phylogenetic tree, we discuss the evolutionary history of the hyper-elongation of the flagellum and associated behaviour.

## 1. Introduction

The hyper-elongation of part of an intromittent organ is widespread in animals, and in some cases intromittent organs can reach lengths several times longer than body length [1,2]. The evolution of such hyper-elongation is widely accepted as the result of sexual selection [3–7] and is accompanied by the evolution of supportive structures and/or behaviours. The insertion of an intromittent organ is mechanically challenging [8,9], and specialized structures or behaviours to increase the manoeuvrability or storage space of intromittent organs have been detected in many insects with a hyper-elongated part of the intromittent organ [2,10–13]. However, evolution occurs via the modification of descendants' phenotypes, which means that organisms cannot always obtain an optimal phenotype from an ancestral state without going through non-optimal intermediate states [14–19]. Nevertheless, many organisms have acquired a hyper-elongated intromittent organ. Here, we describe a case in which a beetle species has overcome the challenge of acquiring a hyper-elongated structure in a unique way.

**Figure 1.**
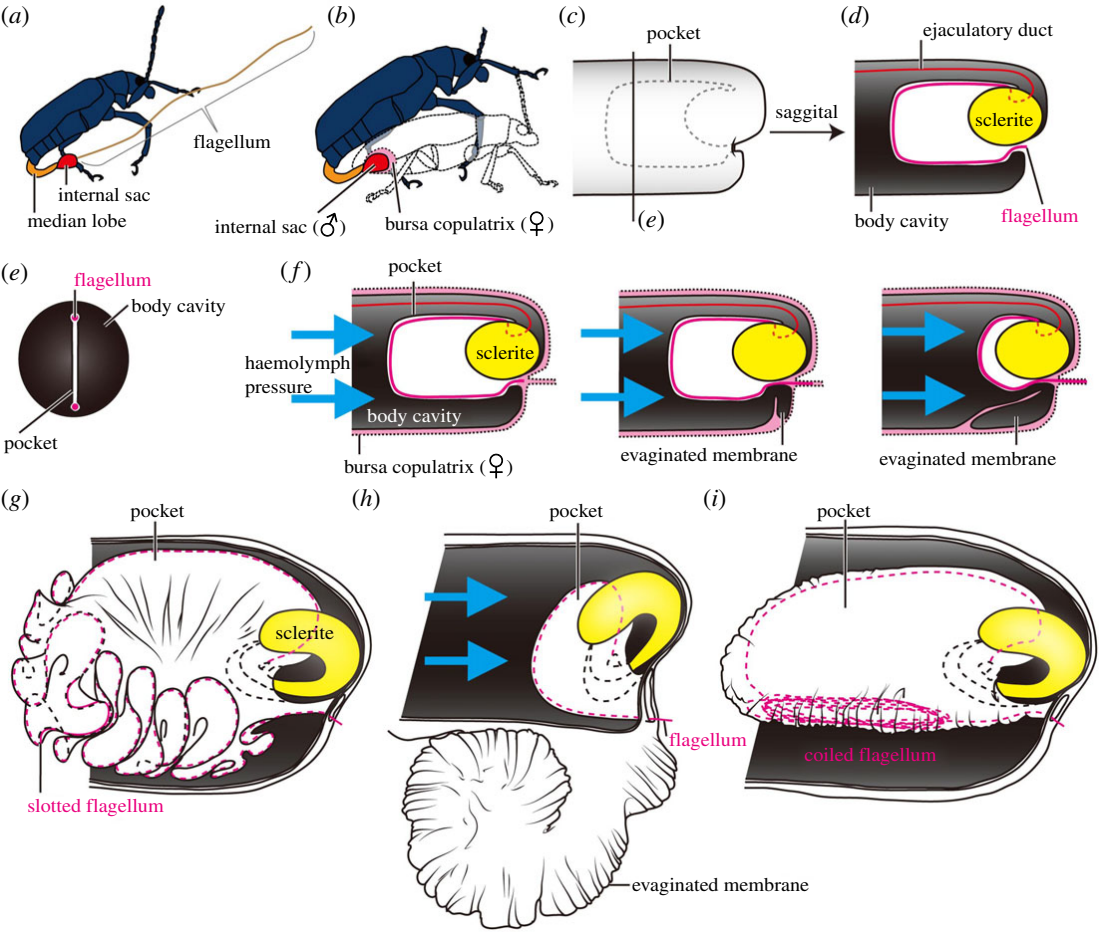
Internal sac morphology at rest and during copulation in *Lema coronata*. (*a*) A scheme showing the relative length of the flagellum. Potentially, this everted position occurs during the eversion and withdrawal process of the internal sac, but the flagellum is too thin to be observed. (*b*) Positional relationship between male and female genitalia during copulation. (*c–e*) Simplified schemes of the internal sac: the apical part is invaginated (dashed line) in the lateral view except in (*e*); (*d*) shows a sagittal view of (*c*); and (*e*) shows a cross-section around the line in (*c*). The pocket is flattened and provides a space for storing the flagellum. (*f*) The flagellar insertion mechanism achieved by haemolymph pressure; due to the presence of sclerites, the pocket is evaginated only from the lower part during the flagellar insertion. (*g–i*) Realistic schemes of the internal sac: in the internal sac found in the field during the reproductive season (*g*), the flagellum is slotted along the strongly undulating margin of the pocket; (*h*) after the pocket is evaginated; (*i*) a male immediately after eclosion, with the flagellum coiled in the pocket.

**Figure 2.**
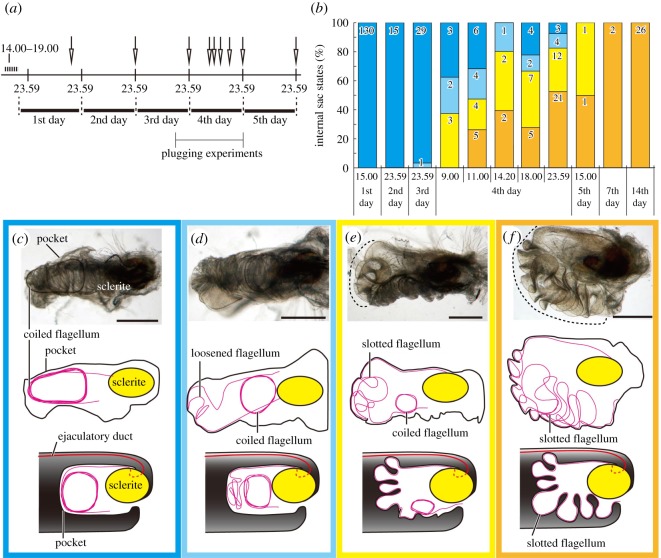
Flagellar position changes in *L. coronata*. (*a*) Time schedule of the current study and definition of ‘days’ after eclosion. We accessed pupal cages daily at approximately 14.00 to 19.00 to separate the newly emerged adults. For simplicity, the 1st day was defined as ending at 23.59 on the next day. Arrows indicate the time at which we fixed the animals. (*b*) Flagellar position changes over time in *L. coronata*. The column colours are congruent with those in panels (*c–f*). (*c–f*) The pocket and flagellum: (*c*) immediately after emergence, the flagellum is completely coiled; (*d*) the flagellum is partly coiled and partly convoluted; (*e*) coils partly remain, but the flagellum is partly slotted in the undulating pocket; (*f*) the state observed in individuals during the reproductive season, with the completely slotted flagellum in the undulating pocket. In the middle row, the outlines of the pocket except for undulating pocket outlines, flagella and the positions of the sclerites, were traced from the upper images. Although in reality the undulating pocket shows a complicated three-dimensional shape, in the lowest row, this shape is depicted as two-dimensional undulating patterns for simplicity. Scale bars: 0.25 mm.

The main species studied here is *Lema coronata*, which has an elongated element (known as a flagellum) as a part of the intromittent organ that is approximately twice the length of the beetle body (flagellar length: ca. 10.4 mm) and very thin (flagellar diameter: less than 2 µm) [20] (figure 1*a*). The anatomy of the intromittent organ is elaborate [21]. In the resting position, the flagellum is stored in an internal sac (figure 1*a*,*b*, red-coloured structure). During copulation the thin flagellum is everted and inserted into a duct of the female's sperm receptacle organ, the spermatheca [21]. The membranous internal sac of the intromittent organ is first evaginated from the median lobe and inserted into a region of the female vagina called the bursa copulatrix [21] (figure 1*b*). The insertion and withdrawal of the flagellum are accomplished quickly due to the very complex configuration of the flagellum and the internal sac membrane, which has been observed only in species of the subgenus *Lema* [11,21]. In the resting position, an apical part of the internal sac membrane is invaginated (figure 1*c*,*d*), the invaginated membrane (hereafter called the pocket) of the internal sac is flattened on the sagittal plane (figure 1*e*), and the flagellum is slotted along the fold [21] (this position is hereafter referred to as the active state; figure 1*c*,*d*). Since the entire flagellum, which is twice as long as the body, is stored in the small internal sac (figure 1*a*), the marginal area of the pocket is strongly enlarged and has an undulating appearance regardless of whether the flagellum is located in it ([21]; Y.M. 2010, personal observation) (figures 1*g* and 2*f*, dashed line). No muscles are directly attached to the flagellum [21]. However, due to the tight relationship between the flagellum and the pocket, the beetle can move and precisely control the flagellum with the eversion of the pocket by adjusting the haemolymph pressure [21] (figure 1*f*,*h*). Nevertheless, this tight relationship between the pocket and the flagellum is absent in newly eclosed adults. In newly eclosed adults, the flagellum is coiled up in the pocket (hereafter referred to as the inactive state), as we describe for the first time in the present paper (figures 1*i* and 2*c*). Previously, it was reported that *Lema coronata* males push the intromittent organ out of the abdomen and evert the internal sac in the absence of females (a video is available in [22]). We thus hypothesized that males uncoil the flagellum before copulation to prepare the intromittent organ for transferring sperm.

To test this hypothesis and identify the factors contributing to acquisition of the active state of the hyper-elongated flagellum, we investigated (i) the stage at which the flagellar location changes (before or after sexual maturation) and (ii) the mechanical uncoiling of the flagellum and the pocket in *L. coronata*. Based on our present observations and reports of morphogenesis by Matsumura *et al*. [23], we discuss why males have the inactive state during morphogenesis. In addition, we investigated the evolution of the characteristic of flagellar uncoiling by comparison among *Lema* species with various flagellar lengths. Based on an associated molecular phylogenetic hypothesis [24], we propose an evolutionary history of the elongated flagellum and related behaviour.

## 2. Material and methods

### 2.1. Rearing and fixation of *L. coronata*

In 2010, adults of *Lema coronata* Baly, 1973 were collected in central Japan in early July, during their reproductive season. Fifty-four adults were collected and placed in a plastic bag with their food plant *Commelina communis*, and the bag was stored in an incubator (27 ± 0.5°C, 16 L : 8 D; the light was turned on at 09.00). The eggs laid were reared to adulthood, and the adults were used in the subsequent experiments. Each day (between approximately 14.00 and 19.00), newly emerged adult males were removed and housed together with other males for further experiments (figure 2*a*). The adults were frozen chronologically in a conventional freezer (−20°C) and were dissected to determine the time at which the position of the flagellum was uncoiled into the active state. The fixation schedule is presented in figure 2*a* with arrows. For this experiment 139 males were used, in addition to internal sac conditional information from our previous studies (1-day-old 130 males: Y.M. 2010, unpublished data; 14 days after emergence and after a first copulation: Matsumura & Yoshizawa [21]).

### 2.2. Chronological changes in vas deferens + testis weights

If the presumed functional state of the flagellum is indispensable for the insertion of the flagellum, as discussed in Matsumura & Yoshizawa [21] based on morphology, it is reasonably predicted that flagellar uncoiling occurs prior to sexual maturation. Because the mature sperm are stored in a seminal vesicle (part of the vas deferens) in insects [20], we measured the dried weight of the vasa deferentia + testes of animals of different ages. One-, 3- and 5-day-old virgin males and field-collected males in the reproductive season that had presumably experienced multiple copulations were fixed using FAA fixative (formaldehyde—acetic acid—ethanol solution) for a couple of days or 70% ethanol. Three, seven, five and five specimens were treated for 1-, 3- and 5-day-old virgin males and field-collected males, respectively. Specimens were preserved in 70% ethanol. One pair of vasa deferentia + testes for each individual was dissected under an Olympus SZ60 stereomicroscope (Olympus, Tokyo, Japan), dried in an incubator and stored at 37°C for one night. The sample was then weighed with an Ultramicro Balance SE-2 (Sartorius Japan K. K., Tokyo, Japan). The weight differences between ages were assessed via an analysis of variance (ANOVA) using R v. 3.1.1 [25].

### 2.3. Quantification of internal sac eversion behaviour

The processes of everting and withdrawing the flagellum are accomplished only through internal sac eversion and withdrawal, which is accomplished by altering the haemolymph pressure in the abdomen, according to Matsumura & Yoshizawa [21]. Therefore, we predicted that males would uncoil the flagellum from the inactive state to the active state by repeated eversion and retraction of the internal sac. As a first step in testing this prediction, we quantified the movement of the intromittent organ on the day of and 1 day prior to the rearrangement occurrence (3 and 4 days after eclosion). Eleven (3-day-old) and 29 (4-day-old) individuals were observed, and the same individuals were never assessed on both days.

**Figure 3.**
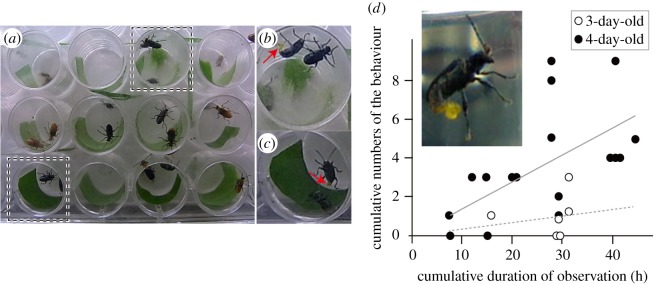
Comparison of the frequency of the internal sac eversion behaviour between the 3- and 4-day-olds after emergence in *L. coronata*. (*a*) The experimental set-up. (*b*,*c*) Enlarged images of males in which the internal sac is everted (arrows) (*b*) at 10.19 and (*c*) at 14.20 on 30 July 2010. (*d*) The results of the experiment, and a male with the internal sac everted.

One or two (in one case, three) males were placed in small cells (1.6 cm in diameter) arranged in a plastic case (12.6 × 8.5 cm) (figure 3*a–c*), and photographs of the beetles in the plastic case were automatically captured at 10-s intervals using a CX1 digital camera (Ricoh, Tokyo, Japan). The males continuously everted their internal sac membranes for more than 10 s in the absence of females (a video is available in Matsumura [22]). Therefore, our non-continuous observation was sufficient to quantify the frequency of the behaviour. The successive pictures of the behaviour thus reflect a series of the behaviour, and the duration of the behaviour was calculated based on the photographs, with each interval equal to 10 s. We recorded the beetles' behaviour for as long as possible, from 00.00 to 24.00. However, because of battery life of the camera being three hours and the inability to record continuously without rest, the observations were fragmented. On average, each cell was observed for 15.1 (3-day-old individuals) and 15.4 (4-day-old individuals) hours (the observation time for each cell is shown in the electronic supplementary material, tables S3 and S5). The number of beetles in a cell varied among cells and the beetles within a cell could not be differentiated in the photos. Therefore, for statistical analyses, we multiplied the duration of the observation of each cell by the number of beetles in each cell and considered this value to be the cumulative observation duration. Then the raw data were summarized into independent six (3-day-old) and 17 (4-day-old) cells, and these were used for the further analysis.

A statistical model to evaluate the effects of age on the frequency of the internal sac eversion behaviour was formulated as a generalized linear mixed model (GLMM), which enabled us to estimate parameters for both fixed effects (in this case, day-age effects) and random effects (the variance between unobservable effects resulting from cell differences). We assumed that the frequency was proportional to the observation time × the number of animals in each cell, so we set the product as an offset term of the GLMM. The Poisson distribution was specified to represent the probability distribution of the observed frequency of the behaviour. The statistical model fittings were performed using R v. 3.1.1 [25] equipped with the glmmML package [26].

### 2.4. Plugging the gateway of the intromittent organ

**Figure 4.**
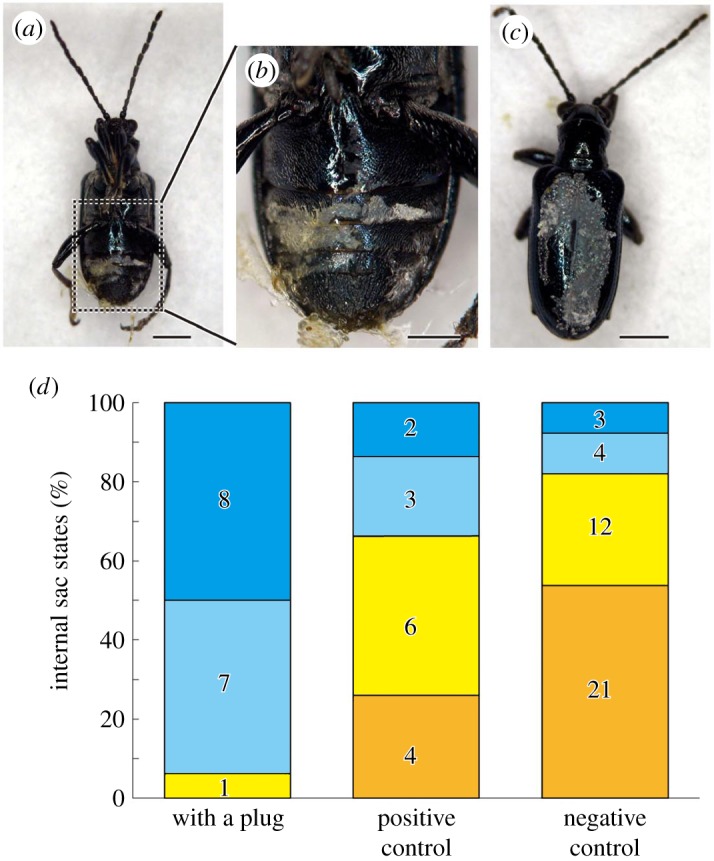
Plugging experiment in *L. coronata.* (*a*) The plugging treatment with the sealed gateway of the intromittent organ in the ventral view and (*b*) enlarged view of the abdomen. (*c*) Positive control treatment, with an individual with glued elytra in the dorsal view. Scale bars indicate 1 mm in (*a*) and (*c*) and 0.5 mm in (*b*). (*d*) Internal sac state frequencies among different treatments. The colours of the columns are congruent with those of figure 2*c–f*: Blue represents the coiled state, and orange represents the functional state.

To test our hypothesis that the rearrangement of the flagellar position into the active state is achieved by eversion of the internal sac, the frequency of flagellar uncoiling into the active state was compared among three treatment groups. In group I, the gateway for the intromittent organ at the abdominal tip was plugged with water-resistant glue (Alon Alpha EX, Komishi, Japan) (figure 4*a*,*b*). In group II, to create a positive control to test the impacts of the super-glue treatment, we applied the super glue to other body parts (figure 4*c*). Three males removed the glue from the gateway soon after the treatment in group I; we treated these animals as members of group II. In group III, the males were left intact as a negative control. The treatment was performed on individuals 1 day prior to the rearrangement (at 22.00 to 23.00 on the 3rd day) under a stereomicroscope, by holding the beetles with tweezers. Then, the beetles were kept with the above mentioned treatments for approximately 24 h and then frozen in a conventional freezer (−20°C) at 24.00 on the day of uncoiling (figure 2*a*). In total, 15 (group I) and 16 (group II) males were analysed, and males fixed at the end of the 4th day after emergence in §2.1 were handled as group III for the statistical analysis. The rearrangement frequencies were compared between the treatments via the *χ*^2^ test with R. v. 3.1.1 [25].

### 2.5. Microstructures on the surface of the pocket

To examine the mechanism of the rearrangement of the flagellum, we scrutinized the surface structure via scanning electron microscopy (SEM). The alcohol-preserved specimens were dehydrated through an ethyl alcohol/t-butyl alcohol series and were freeze-dried. Then, the specimens were coated with palladium and observed using SEM (JSM-5610, JEOL, Japan).

### 2.6. Related species

To evaluate the uniqueness of the rearrangement of the flagellar position and the behaviour observed in *L. coronata*, we explored these features in three closely related species [24]: *Lema dilecta* Baly, 1873, *L. diversa* Baly, 1873, and *L. scutellaris* Kraatz, 1879. These three species have similar body sizes but different flagellar lengths. *Lema coronata* has a flagellar length of 10.4 mm (ca. 1.9-fold its body length). *Lema dilecta* has a flagellar length of 4.20 mm (ca. 1.1-fold), *L*. *diversa* has a flagellar length of 0.74 mm (ca. 0.15-fold), and *L*. *scutellaris* has a flagellar length of 2.03 mm (ca. 0.40-fold) [27]. The data regarding *L. dilecta* were reported as *L. michioi* [27]; however, *L. michioi* is a junior synonym of *L. dilecta* [28].

Quantification of the internal sac eversion behaviour was carried out as mentioned in §2.3. We reared the animals using the methods described above. Due to the difficulty in rearing *L. dilecta*, we used only *L. diversa* and *L. scutellaris* to evaluate behavioural frequencies. Totals of 39 individuals of *L. diversa* and 43 individuals of *L. scutellaris* were used, and all of the males were 4 days old. Because the number of beetles in a cell varied among cells, the raw data were summarized into independent 23 (*L. diversa*) and 26 (*L. scutellaris*) cells and used for the further analysis. The average duration of observation for each cell was 10.8 and 8.3 h for *L. diversa* and *L. scutellaris*, respectively. To compare differences in behavioural frequencies between these two species and *L. coronata*, statistical models (GLMMs, described in §2.4) were fitted to the data. Five logical combinations of species differences were possible. We chose the best combination in terms of goodness-of-prediction by evaluating the Akaike information criterion (AIC).

## 3. Results

All raw and summarized data for the statistical analyses are available in the electronic supplementary material, tables S1–S16.

### 3.1. Chronological changes in flagellar position and testis size in *L. coronata*

Variable conditions of the flagellum in the pocket were observed (figure 2*c–f*). In one condition, the flagellum was in the uncoiled state and completely slotted in the undulating pocket (figure 2*f*). This was the same condition observed in the males collected from the field during the reproductive season (here, we used a laboratory-reared population). In another extreme condition, the flagellum was merely coiled in the middle of an inflated pocket in which folds were recognizable (figures 1*a* and 2*c*), but the flagellum was not slotted into the fold of the pocket (compare figure 2*c* and *f*). Additionally, imperfectly uncoiled and coiled states intermediate between these two conditions were observed. We categorized the intermediate conditions into two categories: (i) the flagellum is partially uncoiled and convoluted (figure 2*d*); (ii) coils are sometimes visible, but the uncoiled flagellum is partly held in the fold of the pocket (figure 2*e*).

In most males, the flagellum was completely coiled until the 3rd day after eclosion (figure 2*b*). The proportion of males exhibiting the active state gradually increased during the 4th day (figure 2*b*). The coiled state was not observed thereafter (figure 2*b*).

**Figure 5.**
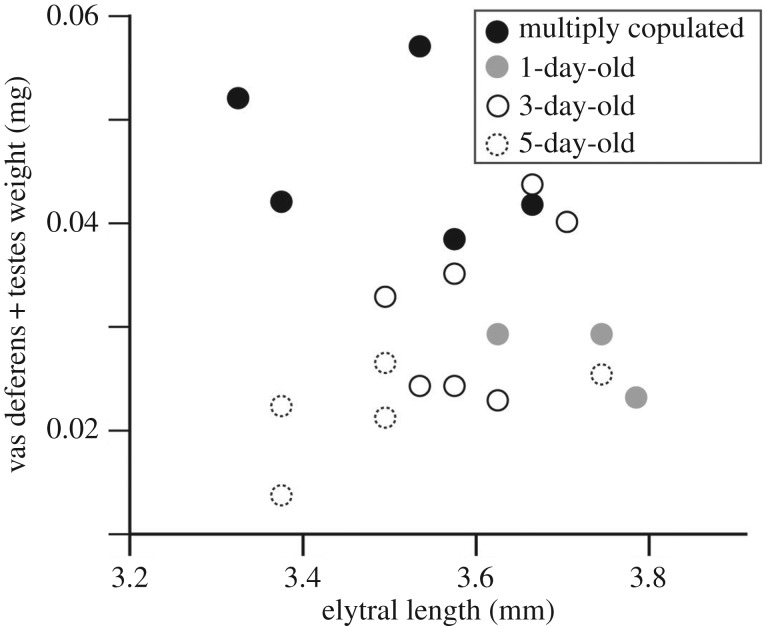
Relationship among the weights of testes + vasa deferentia, elytral lengths and ages in *L. coronata*.

The vas deferens + testis size in males was compared among males in different categories: 1-day-old (*N* = 3), 3-day-old (*N* = 7), 5-day-old (*N* = 5), and presumably after copulation (*N* = 5). It was significantly associated with male age (analysis of covariance (ANCOVA), *F*_3, 16_ = 10.9, *p* < 0.001) but was not significantly influenced by body size (elytral length) (ANCOVA, *F*_1, 15_ = 0.304, *p* = 0.59) (figure 5). The males collected in the field and assumed to have experienced copulation had heavier vasa differentia + testes than other males (figure 5). Therefore, positional changes in the flagellum were completed prior to increasing the weight of the vasa deferentia + testes, which is an indication of sexual maturation.

### 3.2. Eversion and withdrawal of the internal sac in *L. coronata*

The 4-day-old males showed the behaviour more often than did the 3-day-old males. The males repeated series of the behaviour lasting 60.0 ± 20.8 s (means ± standard deviation (s.d.)) (max. 90 s, min. 30 s, 6 times in total) in 3-day-old individuals (*N* = 6) and 73.8 s ± 52.2 (means ± s.d.) (max. 310 s, min. 10 s, 65 times in total) in 4-day-old individuals (*N* = 17). The estimated coefficient of GLMM that represents the differentiation between the 3- and 4-day-olds was 1.40 (standard error (s.e.) 0.427), for which the Wald *p*-value was 0.0011; the 4-day-old males exhibited higher frequencies of this behaviour (figure 3*b*). This was also the case for the duration of the behaviour (estimated coefficient of duration = 1.776, s.e. = 0.570, Wald *p*-value = 0.0019; the duration was estimated using the number of frames exhibiting the behaviour).

### 3.3. Plugging the gateway of the intromittent organ in *L. coronata*

The proportion of males with the active flagellar position and nearly active states was significantly reduced in group I (with the gateway of the intromittent organ plugged) (figure 4*a*,*b*) compared with that in group II (the positive control super-glue-experienced males) (figure 4*c*) and group III (the negative control intact males) (figure 4*d*). In contrast, no differences in frequency were noted between groups II and III (Fisher's exact test: d.f. = 1, *p* = 0.001, for I versus II; d.f. = 1, *p* < 0.0001, I versus III; d.f. = 1, *p* = 0.260, II versus III; *p*-value with Bonferroni correction for multiple comparisons) (figure 4*d*).

### 3.4. Microstructure of the pocket in *L. coronata*

**Figure 6.**
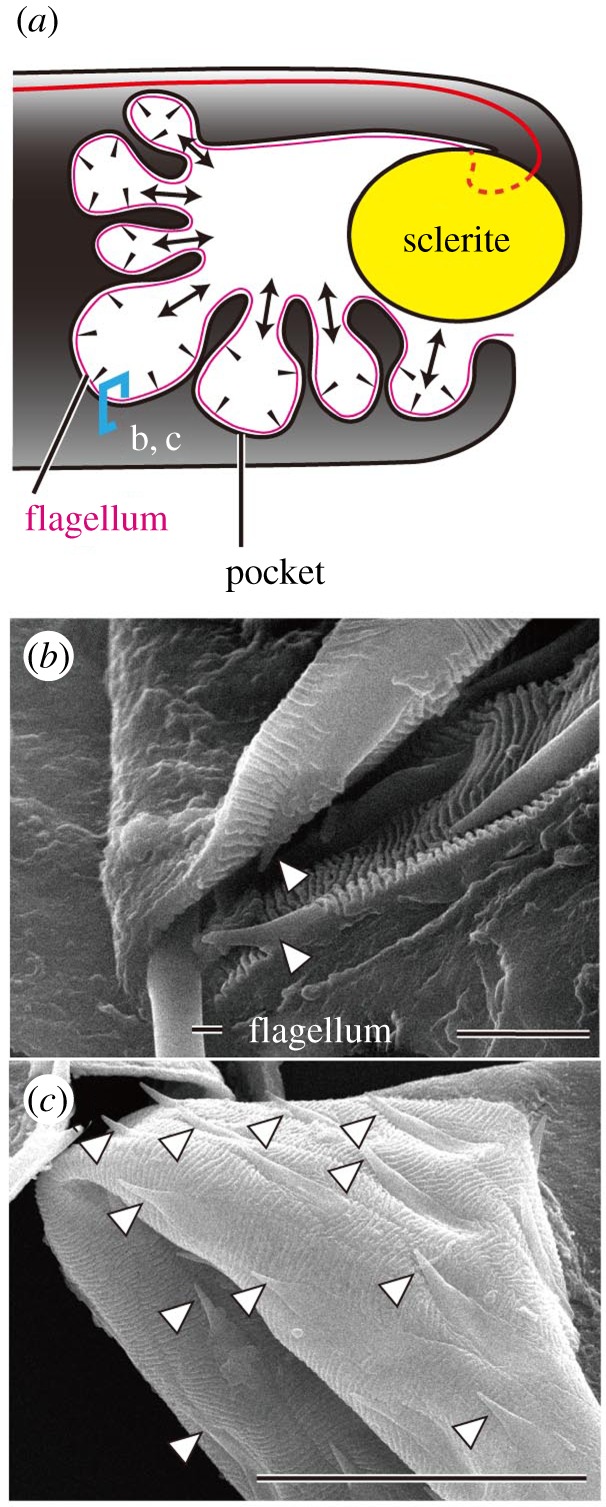
Inner surface structures of the pocket. (*a*) Sagittal view of the internal sac. The black arrowheads indicate the directions of the projections shown in (*b*) and (*c*), and the arrows represent the directions of the reduced friction for flagellar movement (see Discussion). (*b*) A SEM image of the flagellum + pocket, which was torn. (*c*) A SEM image of the pocket, which was torn and everted. Scale bars: 5 µm in (*b*) and 20 µm in (*c*). The white arrowheads point to the spines.

The inner surface of the pocket was covered by fine isotropic wrinkles and spine-like projections (figure 6). The wrinkles were arranged parallel to the flagellum, and the projections were pointed toward the flagellum (figure 6*a*).

### 3.5. Flagellar position change and behaviour in related species

**Figure 7.**
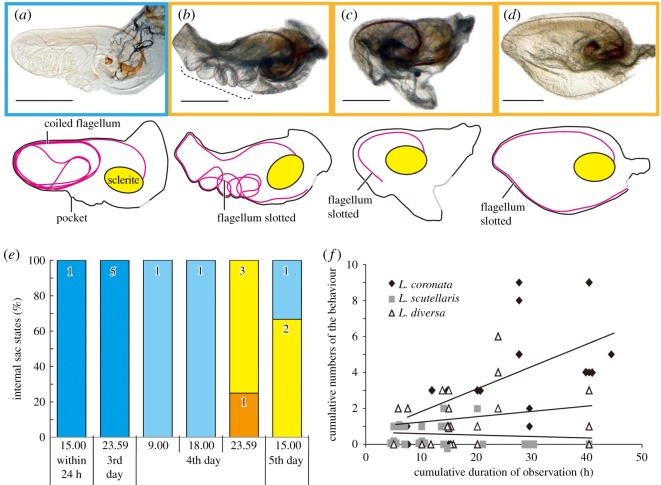
Flagellar position changes in closely related species. (*a–d*) The pocket and flagellum from different species. (*a*) *L. dilecta* immediately after eclosion; the flagellum is completely coiled. (*b*) Idem, the active state observed in the reproductive season; the entire flagellum is grasped by the pocket. (*c*) *Lema diversa* immediately after eclosion. (*d*) *Lema scutellaris* immediately after eclosion. (*e*) Flagellar position changes over time in *L. dilecta*; the colours of the columns are congruent with those of figure 2*c–f*. (*f*) Comparison of the frequency of the internal sac eversion behaviour among species. All individuals were 4 days old. Scale bars: 0.25 mm.

Similar flagellum uncoiling and behaviour were observed in *L. dilecta* and *L. diversa* + *L. scutellaris*, respectively. The flagella of the males at 24 h after eclosion were simply coiled in the middle of the pocket in *L. dilecta* (with a relatively long flagellum) and were slotted in the margin of the pocket in the other species (*L. diversa* and *L. scutellaris*) with relatively short flagella (figure 7*a–d*). Chronologically, the flagellar position in *L. dilecta* underwent changes similar to those in *L. coronata* (figure 7*e*). *Lema diversa* and *L. scutellaris* also exhibited behaviour similar to that in *L. coronata*. *Lema diversa* (*N* = 23) and *L. scutellaris* (*N* = 26) exhibited the behaviour for 41.8 ± 14.9 s (mean ± s.d.) (max. 70 s, min. 20 s, 33 times in total) and 44.6 ± 20.2 s (mean ± s.d.) (max. 70 s, min. 10 s, 13 times in total), respectively. The model assuming that all species were different was selected to be the best (AIC estimate = 101.54). The remaining four AIC estimates were 112.95 (no species differences), 103.76 (*L. coronata* differed) 114.81 (*L. diversa* differed) and 104.45 (*L. scutellaris* differed). The estimated effect sizes for between-species differences were −1.354 (s.e. 0.347) for *L. scutellaris* and −0.617 (s.e. 0.273) for *L. diversa*. Relative to the other species, *Lema coronata* exhibited a higher frequency of the behaviour (figure 7*f*).

## 4. Discussion

The present study shows that the repeated pre-copulatory eversion and withdrawal of the internal sac serves to uncoil the coiled flagellum into the complicated active state in *L. coronata*. The supporting results show that the internal sac eversion behaviour increased on the day when the uncoiling of the flagellar position occurred, i.e. 4 days after eclosion; the absence of this behaviour in 4-day-old adults because of plugging of the genital opening significantly decreased the ratio of those in the active state compared with corresponding ratios in the other treatments. Although the frequency was relatively low, the internal sac eversion behaviour was also observed in 3-day-old adults, when approximately all individuals still had coiled flagella. This finding suggests that a chain of this behaviour alone cannot induce the uncoiling of the flagellum. Males performed the behaviour more than twice on average during the 4th day (we observed only 15 h per individual on average), and many males on the 4th day exhibited intermediate states between the inactive (coiled) and active states. This finding implies that repeated performance of the internal sac eversion behaviour is required for the uncoiling of the flagellum.

How is the flagellum uncoiled by only the eversion and withdrawal of the internal sac? It is conceivable that the spine-like projections on the pocket and isotropic wrinkles facilitate the uncoiling of the flagellum by the repeated eversion of the internal sac. The arrangement of the isotropic wrinkles parallel to the outline of the undulating pocket (figure 6) might decrease the sliding friction between the flagellar and pocket surfaces [29]. This arrangement might decrease the frictional force of the flagellum when it moves in parallel with the undulating margin and make it easier for the flagellum to move toward the undulating margin. Additionally, the projections that are directed toward the outline of the undulating pocket might inhibit the flagellum from moving freely after it aligns with the pocket's outline. Notably, we have never observed the flagellum to slip from the outline in the animals collected from the field during the reproductive season despite hundreds of observations (Y.M. 2010, personal observation).

The flagellum is the only sperm-transferring organ of *L. coronata* [21], which means that the acute control of flagellar penetration is essential for reproduction. In *L. coronata*, the close relationship between the flagellum and pocket enables males to insert and withdraw the long, very thin flagellum with no tangling and minimal breakage during mating [21]. Nevertheless, the present study shows that males emerge with the flagellum in the coiled state, and the positional change of the flagellum into the active position occurs prior to sexual maturation. Therefore, it is likely that the formation of the active state at the end of the morphogenesis of the internal sac is impossible. During morphogenesis, the flagellum and pocket grow simultaneously, and the flagellum grows by extending a fine tube from the flagellar base located at the entrance of the pocket [23]. The growing flagellum is sent into the growing pocket; the flagellum is thus forced to coil in the growing pocket. Without the internal sac eversion behaviour, the beetles cannot uncoil the flagellum into the active state, and this active state is not achieved by morphogenesis alone.

The formation of the coiled flagellum would be correlated with the hyper-elongation of the flagellum in the studied group, subgenus *Lema*. In the related species *L. dilecta* (with a flagellar length approximately 1.1-fold its body length), newly emerged adults also had coiled flagella. The flagellar position changed over time, and the flagellum was stored in the undulating pocket, as it was in *L. coronata*. In contrast, the flagellum was not coiled in the species with substantially shorter flagella and simpler pockets, suggesting that these species complete the morphogenesis of the pocket and flagellum into the active state without the internal sac eversion behaviour.

**Figure 8.**
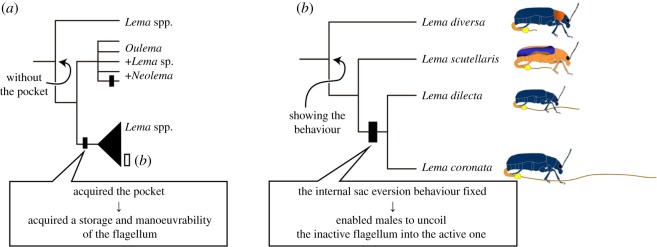
Schematics of the evolutionary history proposed here. The phylogenetic tree was modified from a published study [24]. (*a*) A phylogenetic tree of the *Lema* group; the set of the long flagellum and pocket was acquired at least twice, as indicated by black rectangles [11,24]. (*b*) The phylogenetic relationships among the four species discussed in the present paper and the reconstructed history of the behaviour.

Matsumura & Yoshizawa [11] concluded that the lack of storage space and handling ability of the flagellum hampers the evolution of the hyper-elongated flagellum (figure 8*a*). The intromittent organ is typically stored in a limited space of the abdominal cavity in the Pterygota [30], which confronts insects with challenges regarding the storage and handling of hyper-elongated structures during copulation [2,10,21,31–33]. Therefore, even if beetles can ontogenetically produce the elongated flagellum, the lack of storage space and handling abilities of the precise movement may select against the elongated flagellum. Matsumura & Yoshizawa [11] and Matsumura *et al*. [24] found that in the leaf beetle subfamily Criocerinae, species with a hyper-elongated flagellum evolved only from groups that had acquired the pocket for storing the flagellum. The acquisition of the pocket was essential for the evolution of the hyper-elongated flagellum [11] (figure 8*a*). In addition, based on a phylogenetic hypothesis of these beetles, the behaviour appears to have been preadaptively acquired (figure 8*b*), and the internal sac eversion behaviour was essential for acquiring the hyper-elongated flagellum.

Similar behaviour has been observed in males of the alticine leaf beetle *Altica cyanea* [34] and in female lucanid beetles [35]. These observations imply that a similar phenomenon may be found in other animals, and the function of this behaviour warrants study. Moreover, the unusual behaviour documented here is not only the case in the currently studied species. For example, a rove beetle species with a hyper-elongated flagellum shows specialized behaviour in which the flagellum is guided during withdrawal into the male body cavity [10]. The hyper-elongation of intromittent organs is typically investigated in the context of sexual selection. However, for a comprehensive understanding of the evolution of extremely modified structures, studies of the mechanical challenges that must be overcome in this evolution are also important, as demonstrated by the current study and by Gack & Peschke [10].

## Supplementary Material

Table S1: Raw data from chronological changes of the flagellum in Lema coronata . Emergence means the time we found the eclosed adults.; Table S2. Raw data of vasa deferentia + testes weight (mg) and body size (elytral lengths).; Table S3. Raw data of quantification of the eversion and withdrawal of the internal sac in Lema coronata (3-day-old), observed periods. Unit: min.; Table S4. Raw data of quantification of the eversion and withdrawal of the internal sac in Lema coronata (3-day-old), the periods in which we the behavior was observed. Unit: sec. Individuals in each cell were not distinguishable.; Table S5. Raw data of quantification of the eversion and withdrawal of the internal sac in Lema coronata (4-day-old), observed period. Unit: min.

## Data accessibility

The raw data from the current study have been uploaded as the electronic supplementary material, tables S1–S16; all photographs taken for the behavioural analyses are available on Dryad (http://www.datadryad.org/) at http://dx.doi.org/10.5061/dryad.887g1 [36].
