# Supplementary material for: Eversion and withdrawal of an intromittent organ before sexual maturation prepares male beetles for copulation
Source: R Soc Open Sci. 2017 Aug 9;4(8):161029. doi: 10.1098/rsos.161029 (PMC5579075; doi:10.1098/rsos.161029)
Supplement: Table S1: Raw data from chronological changes of the flagellum in Lema coronata . Emergence means the time we found the eclosed adults.; Table S2. Raw data of vasa deferentia + testes weight (mg) and body size (elytral lengths).; Table S3. Raw data of quantification of the eversion and withdrawal of [file rsos161029supp1.pdf]

Supplementary tables

Table S1. Raw data from chronological changes of the flagellum in *Lema coronata*. Emergence means the time we found the eclosed adults. The data is summerized in Fig. 2b.

1) data from Y. Matsumura unpublished data 2008; 2) the exact emergence date was not memorized in a note book; 3) data from Matsumura & Yoshizawa 2010 (after copulation).

Individuals Emergence		1st day	2nd day	3rd day	4th day					5th day	7th day	14th day
year.month.day	time	-	23:59	23:59	09:00	11:00	14:20	18:00	23:59	-	-	-
1)		coiled										
100722	15-16		coiled									
100722	15-16		coiled									
100722	15-16		coiled									
100722	15-16		coiled									
100722	15-16		coiled									
100722	15-16		coiled									
100722	15-16		coiled									
100722	15-16		coiled									
100722	15-16		coiled									
100723	15-16		coiled									
100723	15-16		coiled									
100723	15-16		coiled									
100723	15-16		coiled									
100723	15-16		coiled									
100723	15-16		coiled									
102724	15-16			coiled								
102724	15-16			coiled								
102724	15-16			coiled								
102724	15-16			coiled								
102724	15-16			coiled								
102724	15-16			coiled								
102724	15-16			coiled								
102724	15-16			coiled								
102724	15-16			coiled								
102724	15-16			coiled								
102724	15-16			coiled								
102725	15-16			coiled								
102725	15-16			coiled								
102725	15-16			coiled								
102725	15-16			coiled								
102725	15-16			coiled								
100725	15-16					partly coiled						
100725	15-16					partly coiled						
100725	15-16					coiled						
100725	15-16					coiled						
100725	15-16					coiled						
100725	15-16					coiled						
100725	15-16					partly coiled						
100725	15-16					functional						
100725	15-16							nearly functional				
100725	15-16							nearly functional				
100725	15-16							nearly functional				
100725	15-16							functional				
100725	15-16								nearly functional			
100725	15-16								functional			
100725	15-16								functional			
100725	15-16								functional			

Table S1 continuous. Raw data from chronological changes of the flagellum in *Lema coronata*. Emergence means the time we found the eclosed adults. The data is summerized in Fig. 2b.

1) data from Y. Matsumura unpublished data 2008; 2) the exact emergence date was not memorized in a note book; 3) data from Matsumura & Yoshizawa 2010 (after copulation).

Individuals												
Emergence		1st day	2nd day	3rd day	4th day					5th day	7th day	14th day
year.month.day	time	-	23:59	23:59	09:00	11:00	14:20	18:00	23:59	-	-	-
100725	15-16							nearly functional				
100725	15-16							nearly functional				
100725	15-16							coiled				
100725	15-16				coiled							
100725	15-16				coiled							
100726	14-15								nearly functional			
100726	14-15								nearly functional			
100726	14-15								nearly functional			
100726	14-15								nearly functional			
100726	14-15								functional			
100726	14-15								functional			
100726	14-15								functional			
100726	14-15								functional			
100726	14-15								functional			
100726	14-15								partly coiled			
100726	14-15								functional			
100726	14-15								coiled			
100726	14-15								functional			
100726	14-15								coiled			
100726	14-15								coiled			
100727	14-15								functional			
100727	14-15								functional			
100727	14-15								functional			
100727	14-15								functional			
100727	14-15								functional			
100727	14-15								functional			
100727	14-15								functional			
100727	14-15								functional			
100727	14-15								nearly functional			
100727	14-15								nearly functional			
100727	14-15								nearly functional			
100727	14-15							functional				
100727	14-15							functional				
100727	14-15							functional				
100727	14-15							nearly functional				
100727	14-15							partly coiled				
100727	14-15							coiled				
100727	14-15								functional			
100727	14-15								nearly functional			
100727	14-15								nearly functional			
100727	14-15				nearly functional							
100727	14-15				nearly functional							
100727	14-15				nearly functional							
100727	14-15				partly coiled							
100727	14-15				partly coiled							
100727	14-15				coiled							
100728	14-15								partly coiled			
100729	14-15								functional			

Table S1 continuous. Raw data from chronological changes of the flagellum in *Lema coronata*. Emergence means the time we found the eclosed adults. The data is summerized in Fig. 2b.

1) data from Y. Matsumura unpublished data 2008; 2) the exact emergence date was not memorized in a note book; 3) data from Matsumura & Yoshizawa 2010.

Emergence		1st day	2nd day	3rd day	4th day					5th day	7th day	14th day
year.month.day	time	-	23:59	23:59	09:00	11:00	14:20	18:00	23:59	-	-	-
100729	14-15								nearly functional			
100729	14-15								partly coiled			
100729	14-15								functional			
100729	14-15					coiled						
100729	14-15					coiled						
100729	14-15					nearly functional						
100812	14-15								partly coiled			
100730	14-15					functional						
100730	14-15					functional						
100730	14-15					functional						
100730	14-15					functional						
100730	14-15					nearly functional						
100730	14-15					nearly functional						
100730	14-15					nearly functional						
100730	14-15					partly coiled						
100730	14-15							functional				
100730	14-15							nearly functional				
100730	14-15							coiled				
100730	14-15							coiled				
100730	14-15							partly coiled				
100809	14-15			coiled								
100809	14-15			coiled								
100809	14-15			coiled								
100809	14-15			coiled								
100809	14-15			coiled								
100816	2)						coiled					
100816	2)						nearly functional					
100816	2)						nearly functional					
100816	2)						functional					
100817	2)						functional					
100816	2)								nearly functional			
100819	2)			coiled								
100819	2)			coiled								
100819	2)			nearly functional								
100819	2)			coiled								
100819	2)			coiled								
100819	2)			coiled								
100819	2)			coiled								
100819	2)			coiled								
100819	2)			coiled								
unknown 2)										nearly functional		
unknown 2)										functional		
unknown 2)											functional	
unknown 2)											functional	
3)												functional

Table S2. Raw data of vasa deferentia + testes weight (mg) and body size (elytral lengths). Status (1st day - 5th day) indicates the days after emergence. The data is summarized in Fig. 3.

State	Elytral length (mm)	Tes+VD weight (mg)
1st day	3.79	0.023
1st day	3.63	0.029
1st day	3.75	0.029
3rd day	3.54	0.024
3rd day	3.67	0.044
3rd day	3.58	0.035
3rd day	3.71	0.040
3rd day	3.58	0.024
3rd day	3.50	0.033
3rd day	3.63	0.023
5th day	3.38	0.014
5th day	3.50	0.026
5th day	3.50	0.021
5th day	3.75	0.025
5th day	3.38	0.022
after copulation	3.58	0.038
after copulation	3.38	0.042
after copulation	3.67	0.042
after copulation	3.54	0.057
after copulation	3.33	0.052

VD: vasa deferentia

Table S3. Raw data of quantification of the eversion and withdrawal of the internal sac in *Lema coronata* (3-day-old), observed periods in each time slot and each cell. Each cell contains two animals numbered 1 or 2 except for A1 in 100724, which contains only one animal. The raw data is summarized in table S7 (duration of observation) by multipying cumulative time of observation by the number of animals in a cell. The emerged data, cell number, and studied animals correspond to that of table S4. Unit: min.

		Time																									
Emerged date	Cell (alphabet), individual (number)	00:00	01:00	02:00	03:00	04:00	05:00	06:00	07:00	08:00	09:00	10:00	11:00	12:00	13:00	14:00	15:00	16:00	17:00	18:00	19:00	20:00	21:00	22:00	23:00	00:00	01:00	Cumulative time of observation (min)
100724	A1								30	60	33		21	60	60	56	60	56	60	60	56	60	56	60	60	33		881
100724	A2								30	60	33		21	60	60	56	60	56	60	60	56	60	56	60	60	33		881
100724	B1								30	60	33		21	60	60	56	60	56	60	60	56	60	56	60	60	33		881
100724	B2								30	60	33		21	60	60	56	60	56	60	60	56	60	56	60	60	33		881
100724	C1								30	60	33		21	60	60	56	60	56	60	60	56	60	56	60	60	33		881
100724	C2								30	60	33		21	60	60	56	60	56	60	60	56	60	56	60	60	33		881
100725	A1										48	60	49	28	60	60	57	60	57	60	60	55	60	60	56	60	49	939
100725	C1										48	60	49	28	60	60	57	60	57	60	60	55	60	60	56	60	49	939
100725	C2										48	60	49	28	60	60	57	60	57	60	60	55	60	60	56	60	49	939
100725	E1										48	60	49	28	60	60	57	60	57	60	60	55	60	60	56	60	49	939
100725	E2										48	60	49	28	60	60	57	60	57	60	60	55	60	60	56	60	49	939
Total (m)									180	360	438	300	371	500	660	636	645	636	645	660	636	635	636	660	640	498	245	
N	11								6	6	11	5	11	11	11	11	11	11	11	11	11	11	11	11	11	11	5	
Total (h)									3	6	7.3	5	6.2	8.3	11	10.6	10.75	10.6	10.75	11	10.6	10.6	10.6	11	10.7	8.3	4.1	

Table S4. Raw data of quantification of the eversion and withdrawal of the internal sac in *Lema coronata* (3-day-old), the periods in which the behavior was observed. The animals were photographed at 10-sec intervals usings a digital camera, and the the duration of the behavior was calculated based on the photographs, with each interval, equal to 10 sec. Individuals in each cell were not distinguishable. Therefore for further analyses we multipied the cumulative number and cumulative periods of the behavior from each cell by the number of animals in a cell. This is summerized in table S7. The emerged data, cell number, and studied animals correspond to that of table S3. Unit: sec.

		Time																										
Emerged date	Cell (alphabet), individual (number)	00:00	01:00	02:00	03:00	04:00	05:00	06:00	07:00	08:00	09:00	10:00	11:00	12:00	13:00	14:00	15:00	16:00	17:00	18:00	19:00	20:00	21:00	22:00	23:00	00:00	01:00	Cumulative periods of the behavior (sec)	Cumulative number of the behavior
100724	A1																											0	0
100724	A2																											0	0
100724	B1																											0	0
100724	B2																											0	0
100724	C1																								90			90	1
100724	C2																											0	0
100725	A1																									80		80	1
100725	C1																							30				30	1
100725	C2																											0	0
100725	E1																				40			60		60		160	3
100725	E2																											0	0
Total (s)																					40			90	90	140			
N																					1			2	1	2			

Table S5. Raw data of quantification of the eversion and withdrawal of the internal sac in *Lema coronata* (4-day-old), observed periods in each time slot and each cell. Each cell contains usually two animals. The cells B in 100731, E in 100816, A in 100817, E and F in 100729, and G in 100730 contain only one animals. The cell D in 100730 contains three animals. The raw data is summarized in table S8 (duration of observation) by multipying cumulative time of observation by the number of animals in a cell. The emerged data, cell number, and studied animals correspond to that of table S6. Unit: min.

Emerged date	Cell (alphabet), individual (number)	Time																										Cumulative duration of observation
		00:00	01:00	02:00	03:00	04:00	05:00	06:00	07:00	08:00	09:00	10:00	11:00	12:00	13:00	14:00	15:00	16:00	17:00	18:00	19:00	20:00	21:00	22:00	23:00	00:00	01:00	
100731	B1								4	60	12				36	60	60	56	60	56	60	60		56	60	60	19	719
100801	A1										3	60	60	36	60	60	57	60	44	49	60	56	60	56	60	52		833
100801	A2										3	60	60	36	60	60	57	60	44	49	60	56	60	56	60	52		833
100801	B1										3	60	60	36	60	60	57	60	44	49	60	56	60	56	60	52		833
100801	B2										3	60	60	36	60	60	57	60	44	49	60	56	60	56	60	52		833
100801	D1										3	60	60	36	60	60	57	60	44	49	60	56	60	56	60	52		833
100801	D2										3	60	60	36	60	60	57	60	44	49	60	56	60	56	60	52		833
100816	E1	30	60	60	2				9	60	60	37	60	60	24													462
100817	A1	14	60	60	26			6	60	60	56	60	49															451
100817	C1	14	60	60	26			6	60	60	56	60	49															451
100817	C2	14	60	60	26			6	60	60	56	60	49															451
100729	A1		9	60	60	16			47	60	60	60	60	55	60	60	56	60	55	60	60	56	60	60	54	60	25	1213
100729	A2		9	60	60	16			47	60	60	60	60	55	60	60	56	60	55	60	60	56	60	60	54	60	25	1213
100729	B1		9	60	60	16			47	60	60	60	60	55	60	60	56	60	55	60	60	56	60	60	54	60	25	1213
100729	B2		9	60	60	16			47	60	60	60	60	55	60	60	56	60	55	60	60	56	60	60	54	60	25	1213
100729	C1		9	60	60	16			47	60	60	60	60	55	60	60	56	60	55	60	60	56	60	60	54	60	25	1213
100729	C2		9	60	60	16			47	60	60	60	60	55	60	60	56	60	55	60	60	56	60	60	54	60	25	1213
100729	D1		9	60	60	16			47	60	60	60	60	55	60	60	56	60	55	60	60	56	60	60	54	60	25	1213
100729	D2		9	60	60	16			47	60	60	60	60	55	60	60	56	60	55	60	60	56	60	60	54	60	25	1213
100729	E1		9	60	60	16			47	60	60	60	60	55	60	60	56	60	55	60	60	56	60	60	54	60	25	1213
100729	F1		9	60	60	16			47	60	60	60	60	55	60	60	56	60	55	60	60	56	60	60	54	60	25	1213
100730	B1										58	60	39	48	60	57	60	60	49	60	60	56	60	53	48	60	1	889
100730	B2										58	60	39	48	60	57	60	60	49	60	60	56	60	53	48	60	1	889
100730	C1										58	60	39	48	60	57	60	60	49	60	60	56	60	53	48	60	1	889
100730	C2										58	60	39	48	60	57	60	60	49	60	60	56	60	53	48	60	1	889
100730	D1										58	60	39	48	60	57	60	60	49	60	60	56	60	53	48	60	1	889
100730	D2										58	60	39	48	60	57	60	60	49	60	60	56	60	53	48	60	1	889
100730	D3										58	60	39	48	60	57	60	60	49	60	60	56	60	53	48	60	1	889
100730	G1										58	60	39	48	60	57	60	60	49	60	60	56	60	53	48	60	1	889
Total (m)		72	330	840	680	160	0	18	663	900	1322	1657	1479	1210	1500	1476	1442	1496	1266	1430	1500	1404	1440	1416	1344	1452	277	
N	29	4	14	14	14	10	0	3	15	15	29	28	28	25	26	25	25	25	25	25	25	25	24	25	25	25	19	
Total (h)		1.2	5.5	14.0	11.3	2.7	0.0	0.3	11.1	15.0	22.0	27.6	24.7	20.2	25.0	24.6	24.0	24.9	21.1	23.8	25.0	23.4	24.0	23.6	22.4	24.2	4.6	

Table S6. Raw data of quantification of the eversion and withdrawal of the internal sac in *Lema coronata* (4-day-old), the periods in which the behavior was observed. The animals were photographed at 10-sec intervals usings a digital camera, and the the duration of the behavior was calculated based on the photographs, with each interval, equal to 10 sec. Individuals in each cell were not distinguishable. Therefore for further analyses we multipied the cumulative number and cumulative periods of the behavior from each cell by the number of animals in a cell. This is summerized in table S8. The emerged data, cell number, and studied animals correspond to that of table S5. Unit: sec.

Emerged date	Cell (alphabet), individual (number)	Time																										Cumulative duration of the behavior	Cumultive numbers of the behavior
		00:00	01:00	02:00	03:00	04:00	05:00	06:00	07:00	08:00	09:00	10:00	11:00	12:00	13:00	14:00	15:00	16:00	17:00	18:00	19:00	20:00	21:00	22:00	23:00	00:00	01:00		
100731	B1																		40		60		90					190	3
100801	A1												40	50				70		60						90		310	5
100801	A2																												
100801	B1																		70	230	60	140	310	130	230			1170	7
100801	B2																						50		50			100	2
100801	D1														80						110	130		90	110	120		640	6
100801	D2																				70				190	80		340	3
100816	E1																											0	0
100817	A1												90															90	1
100817	C1																											0	0
100817	C2																												
100729	A1											50									70			60			80	260	4
100729	A2																												
100729	B1																					80				70		150	2
100729	B2																					20				100		120	2
100729	C1																		140		10		90		80			320	4
100729	C2																												
100729	D1											60		30			70	80		50	20		100		60	80		550	9
100729	D2																												
100729	E1									60			10													60		130	3
100729	F1												90	60		30												180	3
100730	B1															40										70		110	2
100730	B2																												
100730	C1																					60						60	1
100730	C2																												
100730	D1											50						120	70		190				90			520	5
100730	D2																												
100730	D3																												
100730	G1																		70		70		70					210	3
Total (s)		0	0	0	0	0	0	0	0	60	0	160	230	140	80	70	70	270	390	340	660	430	710	280	810	670	80		
N		0	0	0	0	0	0	0	0	1	0	3	4	3	1	2	1	3	5	3	9	5	6	3	7	8	1		

Table S7. Summarized data of the eversion and withdrawal of the internal sac in 3-day-old *Lema coronata.* The original raw data is shown in tables 3-4. Although we treated 11 animals in 3-day-old, animals in same cells were not distinguishable. Therefore the raw data was analyzed as six differnt samples. The data was used for further analysis showin in Fig. 4b.

Emergence date	Cell	Duration of the behavior (s)	The number of the behavior	Duration of observation (m)	The number of individuals in a cell
100724	A	0	0	29.4	2
100724	B	0	0	29.4	2
100724	C	90	1	29.4	2
100725	A	80	1	15.7	1
100725	C	30	1	31.3	2
100725	E	160	3	31.3	2

Table S8. Summarized data of the eversion and withdrawal of the internal sac in 4-day-old *Lema coronata.* The original raw data is shown in tables 5-6. Although we treated 29 animals in 4-day-old, animals in same cells were not distinguishable. Therefore the raw data was analyzed as 17 differnt samples. The data was used for further analysis showin in Fig. 4b.

Emergence date	Cell	Duration of the behavior (s)	The number of the behavior	Duration of observation (m)	The number of individuals in a cell
100731	B	190	3	12.0	1
100801	A	310	5	27.8	2
100801	B	1270	8	27.8	2
100801	D	980	9	27.8	2
100816	E	0	0	7.7	1
100817	A	90	1	7.5	1
100817	C	0	0	15.0	2
100729	A	260	4	40.4	2
100729	B	270	4	40.4	2
100729	C	320	4	40.4	2
100729	D	550	9	40.4	2
100729	E	130	3	20.2	1
100729	F	180	3	20.2	1
100730	B	110	2	29.6	2
100730	C	60	1	29.6	2
100730	D	520	5	44.5	3
100730	G	210	3	14.8	1

Table S9. Raw data of plugging the gateway experiment in *Lema coronata* . This data corresponts to results of "with a plug" and "positive control" in Fig. 5d. The data was compared with the internal sac state data in the end of 4th day after emergence in table 1 (Fig. 2b) as the negative control.

Individuals						
Emerged date		Treatment	Fixation	States	Treatment	The place glued.
year.month.day	time	3rd day	4th day			
100812	14-15	23:00	23:59	nearly functional	with a plug	opening of the genitalia
100818	14-15	17:30	23:59	coiled	with a plug	opening of the genitalia
100818	14-15	17:30	23:59	coiled	with a plug	opening of the genitalia
100818	14-15	17:30	23:59	partly coiled	with a plug	opening of the genitalia
100818	14-15	17:30	23:59	partly coiled	with a plug	opening of the genitalia, abdomen
100818	14-15	17:30	23:59	partly coiled	with a plug	opening of the genitalia, abdomen
100816	14-15	23:00	23:59	partly coiled	with a plug	opening of the genitalia, abdomen
100816	14-15	23:00	23:59	partly coiled	with a plug	opening of the genitalia, abdomen
100816	14-15	23:00	23:59	coiled	with a plug	opening of the genitalia, abdomen
100816	14-15	23:00	23:59	partly coiled	with a plug	opening of the genitalia, abdomen
100730	14-15	23:00	23:59	partly coiled	with a plug	opening of the genitalia
100730	14-15	23:00	23:59	coiled	with a plug	opening of the genitalia
100730	14-15	23:00	23:59	coiled	with a plug	opening of the genitalia
100730	14-15	23:00	23:59	coiled	with a plug	opening of the genitalia
100730	14-15	23:00	23:59	coiled	with a plug	opening of the genitalia
100730	14-15	23:30	23:59	coiled	with a plug	opening of the genitalia
100815	14-15	23:30	23:59	partly coiled	positive control	eltytra, pronotum
100815	14-15	23:30	23:59	coiled	positive control	eltytra, pronotum
100812	14-15	23:00	23:59	nearly functional	positive control	elytra
100812	14-15	23:00	23:59	functional	positive control	elytra, but removed before fixation
100812	14-15	23:00	23:59	functional	positive control	elytra, but removed before fixation
100818	14-15	17:30	23:59	partly coiled	positive control	the glue was put on the genital opening but removed soon.
100818	14-15	17:30	23:59	nearly functional	positive control	the glue was put on the genital opening but removed soon.
100818	14-15	17:30	23:59	nearly functional	positive control	legs
100818	14-15	17:30	23:59	functional	positive control	legs
100818	14-15	17:30	23:59	nearly functional	positive control	elytra
100818	14-15	17:30	23:59	coiled	positive control	elytra
100818	14-15	17:30	23:59	nearly functional	positive control	legs
100816	14-15	23:00	23:59	nearly functional	positive control	elytra
100816	14-15	23:00	23:59	functional	positive control	elytra
100730	14-15	23:00	23:59	partly coiled	positive control	the glue was put on the genital opening but removed soon.

Table S10. Raw data from chronological positional changing of the flagellum in *Lema dilecta* . This data is summerized in Fig. 7e. Emergence means the time we found the hatched adults. 1) the exact emergence date was not memorized in a note book.

Emergence		1st day	3rd day	4th day			5th day
year.month.day	time	23:59	23:59	09:00	18:00	23:59	15:30
100724	15-16		coiled				
100724	15-16		coiled				
100724	15-16		coiled				
100724	15-16		coiled				
100724	15-16		coiled				
100828	1)					functional	
100727	1)			partly coiled			
100730	1)				partly coiled		
110727	1)					nearly functional	
110728	1)					nearly functional	
110731	1)						partly coiled
110731	1)						nearly functional
110731	1)						nearly functional
100804	1)	coiled					
100729	1)					nearly functional	

Table S11. Raw data of quantification of the eversion and withdrawal of the internal sac in *Lema diversa* (4-day -old), observed periods in each time slot and each cell. Each cell contains usually two animals numbered 1 or 2, except for the cells C in 100801, - in 100808, A in 100810, A in 100809, L in 100729, and B in 100730, which contain only one animal. The raw data is summarized in table S15 (cumultative periods of observation) by multipying cumulative periods of observation by the number of animals in a cell. The emerged data, cell number, and studied animals correspond to that of table S13. Unit: min.

Emerged date		Time																									
year.month. day	cell (alphabet), individial (number)	00:00	01:00	02:00	03:00	04:00	05:00	06:00	07:00	08:00	09:00	10:00	11:00	12:00	13:00	14:00	15:00	16:00	17:00	18:00	19:00	20:00	21:00	22:00	23:00	00:00	01:00	Cumulative periods of observation
100731	A1								4	60	12				36	60	60	56	60	56	60	60		56	60	60	19	719
100731	A2								4	60	12				36	60	60	56	60	56	60	60		56	60	60	19	719
100731	C1								4	60	12				36	60	60	56	60	56	60	60		56	60	60	19	719
100731	C2								4	60	12				36	60	60	56	60	56	60	60		56	60	60	19	719
100731	D1								4	60	12				36	60	60	56	60	56	60	60		56	60	60	19	719
100731	D2								4	60	12				36	60	60	56	60	56	60	60		56	60	60	19	719
100801	C1										3	60	60	36	60	60	57	60	44	49	60	56	60	56	60	52		833
100808	-	59	60	37			7	60	60	55	60	60																458
100810	A	3	60	38					15	60	60	54	60	4														354
100810	B1	3	60	38					15	60	60	54	60	4														354
100810	B2	3	60	38					15	60	60	54	60	4														354
100809	A1	50	60	41						35	60	59																305
100809	B1	50	60	41						35	60	59																305
100809	B2	50	60	41						35	60	59																305
100816	A1	30	60	60	2				9	60	60	37	60	60	24													462
100816	A2	30	60	60	2				9	60	60	37	60	60	24													462
100816	B1	30	60	60	2				9	60	60	37	60	60	24													462
100816	B2	30	60	60	2				9	60	60	37	60	60	24													462
100816	C1	30	60	60	2				9	60	60	37	60	60	24													462
100816	C2	30	60	60	2				9	60	60	37	60	60	24													462
100816	D1	30	60	60	2				9	60	60	37	60	60	24													462
100816	D2	30	60	60	2				9	60	60	37	60	60	24													462
100817	B1	14	60	60	26			6	60	60	56	60	49															451
100817	B2	14	60	60	26			6	60	60	56	60	49															451
100817	D1	14	60	60	26			6	60	60	56	60	49															451
100817	D2	14	60	60	26			6	60	60	56	60	49															451
100817	E1	14	60	60	26			6	60	60	56	60	49															451
100817	E2	14	60	60	26			6	60	60	56	60	49															451
100817	F1	14	60	60	26			6	60	60	56	60	49															451
100817	F2	14	60	60	26			6	60	60	56	60	49															451
100729	G1		9	60	60	16			47	60	60	60	60	55	60	60	56	60	55	60	60	56	60	60	54	60	25	1213
100729	I1		9	60	60	16			47	60	60	60	60	55	60	60	56	60	55	60	60	56	60	60	54	60	25	1213
100729	I2		9	60	60	16			47	60	60	60	60	55	60	60	56	60	55	60	60	56	60	60	54	60	25	1213
100729	J1		9	60	60	16			47	60	60	60	60	55	60	60	56	60	55	60	60	56	60	60	54	60	25	1213
100729	J2		9	60	60	16			47	60	60	60	60	55	60	60	56	60	55	60	60	56	60	60	54	60	25	1213
100729	K1		9	60	60	16			47	60	60	60	60	55	60	60	56	60	55	60	60	56	60	60	54	60	25	1213
100729	K2		9	60	60	16			47	60	60	60	60	55	60	60	56	60	55	60	60	56	60	60	54	60	25	1213
100729	L1		9	60	60	16			47	60	60	60	60	55	60	60	56	60	55	60	60	56	60	60	54	60	25	1213
100730	E1										58	60	39	48	60	57	60	60	49	60	60	56	60	53	48	60	1	889
Total (m)		570	1452	1714	704	128	7	108	1057	2140	1961	1775	1631	1016	1008	957	925	936	893	925	960	920	600	925	900	952	315	
N		23	31	31	24	8	1	9	34	37	39	33	29	21	24	16	16	16	16	16	16	16	10	16	16	16	15	
Total (h)		9.5	24.2	28.6	11.7	2.1	0.1	1.8	17.6	35.7	32.7	29.6	27.2	16.9	16.8	16.0	15.4	15.6	14.9	15.4	16.0	15.3	10.0	15.4	15.0	15.9	5.3	

Table S12. Raw data of quantification of the eversion and withdrawal of the internal sac in *Lema scutellaris* (4-day -old)), observed periods in each time slot and each cell. Individuals in each cell were not distinguishable. Each cell contains usually two animals numbered 1 or 2, except for the cells C in 100810, D in 100810, C in 100809, D in 100809, A in 100812, B in 100812, H in 100812, G in 100814, H in 100814, and G in 100729, which contain only one animal. The raw data is summarized in table S16 (cumultative periods of observation) by multipying cumulative periods of observation by the number of animals in a cell. The emerged data, cell number, and studied animals correspond to that of table S14. Unit: min.

Emerged date		Time																										
year.month. day	cell (alphabet), individial (number)	00:00	01:00	02:00	03:00	04:00	05:00	06:00	07:00	08:00	09:00	10:00	11:00	12:00	13:00	14:00	15:00	16:00	17:00	18:00	19:00	20:00	21:00	22:00	23:00	00:00	01:00	Cumulative periods of observation (min)
100810	C1	3	60	38					15	60	60	54	60	4														354
100810	D1	3	60	38					15	60	60	54	60	4														354
100809	C1	50	60	41						35	60	59																305
100809	D1	50	60	41						35	60	59																305
100812	A1	52	60	44						10	60	60	18															304
100812	B1	52	60	44						10	60	60	18															304
100812	C1	52	60	44						10	60	60	18															304
100812	C2	52	60	44						10	60	60	18															304
100812	D1	52	60	44						10	60	60	18															304
100812	D2	52	60	44						10	60	60	18															304
100812	E1	52	60	44						10	60	60	18															304
100812	E2	52	60	44						10	60	60	18															304
100812	F1	52	60	44						10	60	60	18															304
100812	F2	52	60	44						10	60	60	18															304
100812	G1	52	60	44						10	60	60	18															304
100812	G2	52	60	44						10	60	60	18															304
100812	H1	52	60	44						10	60	60	18															304
100814	A1	52	60	42						56	60	56	60	38														424
100814	A2	52	60	42						56	60	56	60	38														424
100814	B1	52	60	42						56	60	56	60	38														424
100814	B2	52	60	42						56	60	56	60	38														424
100814	C1	52	60	42						56	60	56	60	38														424
100814	C2	52	60	42						56	60	56	60	38														424
100814	D1	52	60	42						56	60	56	60	38														424
100814	D2	52	60	42						56	60	56	60	38														424
100814	E1	52	60	42						56	60	56	60	38														424
100814	E2	52	60	42						56	60	56	60	38														424
100814	F1	52	60	42						56	60	56	60	38														424
100814	F2	52	60	42						56	60	56	60	38														424
100814	F3	52	60	42						56	60	56	60	38														424
100814	G1	52	60	42						56	60	56	60	38														424
100814	H1	52	60	42						56	60	56	60	38														424
100814	J1	52	60	42						56	60	56	60	38														424
100814	J2	52	60	42						56	60	56	60	38														424
100729	G1		9	60	60	16			47	60	60	60	60	55	60	60	56	60	55	60	60	56	60	60	54	60	25	1213
100729	H1		9	60	60	16			47	60	60	60	60	55	60	60	56	60	55	60	60	56	60	60	54	60	25	1213
100729	H2		9	60	60	16			47	60	60	60	60	55	60	60	56	60	55	60	60	56	60	60	54	60	25	1213
100730	A1										58	60	39	48	60	57	60	60	49	60	60	56	60	53	48	60	1	889
100730	A2										58	60	39	48	60	57	60	60	49	60	60	56	60	53	48	60	1	889
100730	F1										58	60	39	48	60	57	60	60	49	60	60	56	60	53	48	60	1	889
100730	F2										58	60	39	48	60	57	60	60	49	60	60	56	60	53	48	60	1	889
100730	H1										58	60	39	48	60	57	60	60	49	60	60	56	60	53	48	60	1	889
100730	H2										58	60	39	48	60	57	60	60	49	60	60	56	60	53	48	60	1	889
Total (m)		1666	2067	1624	180	48	0	0	171	1452	2568	2498	1788	1107	540	522	528	540	459	540	540	504	540	498	450	540	81	
N		34	37	37	3	3	0	0	5	37	43	43	41	28	9	9	9	9	9	9	9	9	9	9	9	9	9	
Total (h)		27.8	34.5	27.1	3.0	0.8	0.0	0.0	2.9	24.2	42.8	41.6	29.8	18.5	9.0	8.7	8.8	9.0	7.7	9.0	9.0	8.4	9.0	8.3	7.5	9.0	1.4	

Table S13. Raw data of quantification of the eversion and withdrawal of the internal sac in *Lema diversa* (4-day -old), the periods in which the behavior was observed. The animals were photographed at 10-sec intervals usings a digital camera, and the the duration of the behavior was calculated based on the photographs, with each interval, equal to 10 sec. Individuals in each cell were not distinguishable. Therefore for further analyses we multipied the cumulative number and cumulative periods of the behavior from each cell by the number of animals in a cell. This is summerized in table S15. The emerged data, cell number, and studied animals correspond to that of table S11. Unit: sec.

Emerged date		Time																											
year.month. day	cell (alphabet), individial (number)	00:00	01:00	02:00	03:00	04:00	05:00	06:00	07:00	08:00	09:00	10:00	11:00	12:00	13:00	14:00	15:00	16:00	17:00	18:00	19:00	20:00	21:00	22:00	23:00	00:00	01:00	Cumulated periodes of the behavior (sec)	Cumulated numbers of the behavior
100731	A1																	30				30	70	60				190	4
100731	A2																											0	0
100731	C1															30			50			20	40		50			190	5
100731	C2																					30						30	1
100731	D1																		30						30			60	2
100731	D2																											0	0
100801	C1														50			20, 60										50	3
100808	-								40			30																70	2
100810	A									60	70																	130	2
100810	B1																											0	0
100810	B2																											0	0
100809	A1																											0	0
100809	B1																											0	0
100809	B2																											0	0
100816	A1																											0	0
100816	A2																											0	0
100816	B1																											0	0
100816	B2																											0	0
100816	C1																											0	0
100816	C2																											0	0
100816	D1										50																	50	1
100816	D2																											0	0
100817	B1												40															40	1
100817	B2																											0	0
100817	D1								50	40																		90	2
100817	D2																											0	0
100817	E1									60																		60	1
100817	E2																											0	0
100817	F1								40	30		40																110	3
100817	F2																											0	0
100729	G1																											0	0
100729	I1										20																	20	1
100729	I2																											0	0
100729	J1												50													70		120	2
100729	J2												40															40	1
100729	K1																											0	0
100729	K2																											0	0
100729	L1																										20	20	1
100730	E1																		30									30	1
Total (s)		0	0	0	0	0	0	0	130	190	140	70	130	0	50	30	0	110	110	0	0	80	110	60	80	70	20		
N	39	0	0	0	0	0	0	0	3	4	3	2	3	0	1	1	0	3	3	0	0	3	2	1	2	1	1		

Table S14. Raw data of quantification of the eversion and withdrawal of the internal sac in *Lema scutellaris* (4-day -old), the periods in which the behavior was observed. The animals were photographed at 10-sec intervals usings a digital camera, and the the duration of the behavior was calculated based on the photographs, with each interval, equal to 10 sec. Individuals in each cell were not distinguishable. Therefore for further analyses we multipied the cumulative number and cumulative periods of the behavior from each cell by the number of animals in a cell. This is summerized in table S16. The emerged data, cell number, and studied animals correspond to that of table S12. Unit: sec.

Emerged date		Time																											
year.month. day	cell (alphabet), individial (number)	00:00	01:00	02:00	03:00	04:00	05:00	06:00	07:00	08:00	09:00	10:00	11:00	12:00	13:00	14:00	15:00	16:00	17:00	18:00	19:00	20:00	21:00	22:00	23:00	00:00	01:00	Cumulative periods of the behavior (sec)	Cumulative numbers of the behavior
110810	C1									40																		40	1
110810	D1									50																		50	1
110809	C1									40																		40	1
110809	D1																											0	0
110812	A1																											0	0
110812	B1																											0	0
110812	C1												70															70	1
110812	C2																											0	0
110812	D1																											0	0
110812	D2																											0	0
110812	E1																											0	0
110812	E2																											0	0
110812	F1																											0	0
110812	F2																											0	0
110812	G1																											0	0
110812	G2																											0	0
110812	H1																											0	0
110814	A1										70	50																120	2
110814	A2																											0	0
110814	B1												10															10	1
110814	B2												50															50	1
110814	C1																											0	0
110814	C2																											0	0
110814	D1																											0	0
110814	D2																											0	0
110814	E1										60																	60	1
110814	E2																											0	0
110814	F1																											0	0
110814	F2																											0	0
110814	F3																											0	0
110814	G1										70																	70	1
110814	H1																											0	0
110814	J1												20															20	1
110814	J2																											0	0
110729	G1														10				40									50	2
110729	H1																											0	0
110729	H2																											0	0
100730	A1																											0	0
100730	A2																											0	0
100730	F1																											0	0
100730	F2																											0	0
100730	H1																											0	0
100730	H2																											0	0
Total (s)		0	0	0	0	0	0	0	0	130	200	50	150	0	10	0	0	0	40	0	0	0	0	0	0	0	0		
N	43	0	0	0	0	0	0	0	0	3	3	1	4	0	1	0	0	0	1	0	0	0	0	0	0	0	0		

Table S15. Analyzed data of qunatification of the eversion and withdrawal of the internal sac in 4-day-old *Lema diversa* . The original raw data is shown in tables 11 and 13. Although we treated 39 animals in 4-day-old, animals in same cells were not distinguishable. Therefore the raw data was analyzed as 23 differnt samples listed below. The data was used for further analysis showin in Fig. 7f.

Emerged date	Cell	Cumulative periods of the behavior observed	Cumulative number of the behavior	Cumulative periods of obsevation (h)	The number of individuals in a cell
100731	A	190	4	24.0	2
100731	C	220	6	24.0	2
100731	D	60	2	24.0	2
100801	C	50	3	13.9	1
100808	-	70	2	7.6	1
100810	A	130	2	5.9	1
100810	B	0	0	11.8	2
100809	A	0	0	5.1	1
100809	B	0	0	10.2	2
100816	A	0	0	15.4	2
100816	B	0	0	15.4	2
100816	C	0	0	15.4	2
100816	D	50	1	15.4	2
100817	B	40	1	15.0	2
100817	D	90	2	15.0	2
100817	E	60	1	15.0	2
100817	F	110	3	15.0	2
100729	G	0	0	20.2	1
100729	I	20	1	40.4	2
100729	J	160	3	40.4	2
100729	K	0	0	40.4	2
100729	L	20	1	20.2	1
100730	E	30	1	14.8	1

Table S16. Analyzed data of qunatification of the eversion and withdrawal of the internal sac in 4-day-old *Lema scutellaris* . The original raw data is shown in tables 12 and 14. Although we treated 43 animals in 4-day-old, animals in same cells were not distinguishable. Therefore the raw data was analyzed as 26 differnt samples listed below. The data was used for further analysis showin in Fig. 7f.

Emerged date	Cell	Cumulative periods of the behavior observed	Cumulative number of the behavior	Cumulative periods of obsevation (h)	The number of individuals in a cell
100810	C	40	1	5.9	1
100810	D	50	1	5.9	1
100809	C	40	1	5.1	1
100809	D	0	0	5.1	1
100812	A	0	0	5.1	1
100812	B	0	0	5.1	1
100812	C	70	1	10.1	2
100812	D	0	0	10.1	2
100812	E	0	0	10.1	2
100812	F	0	0	10.1	2
100812	G	0	0	10.1	2
100812	H	0	0	5.1	1
100814	A	120	2	14.1	2
100814	B	60	2	14.1	2
100814	C	0	0	14.1	2
100814	D	0	0	14.1	2
100814	E	60	1	14.1	2
100814	F	0	0	21.2	3
100814	G	70	1	7.1	1
100814	H	0	0	7.1	1
100814	J	20	1	14.1	2
100729	G	50	2	20.2	1
100729	H	0	0	40.4	2
100730	A	0	0	29.6	2
100730	F	0	0	29.6	2
100730	H	0	0	29.6	2
